# Intravenous Immunoglobulin and Intravenous Acyclovir as an Alternative Therapy to Varicella Zoster Immunoglobulin in the Prevention of Serious Complications of Neonatal Varicella

**DOI:** 10.7759/cureus.63515

**Published:** 2024-06-30

**Authors:** Ayman A Alhwayan, Aseel Alsallal, Mohammad Njadat, Malek Alhammad, Baha Haddadin

**Affiliations:** 1 Hematology and Oncology, Jordan Armed Forces Royal Medical Services/Queen Rania Children’s Hospital, Amman, JOR; 2 Pediatrics and Neonatology, Jordan Armed Forces Royal Medical Services, Amman, JOR

**Keywords:** acyclovir, varicella-zoster virus, varicella zoster immunoglobulin, intravenous immunoglobulin, neonatal varicella

## Abstract

Neonatal varicella, arising from maternal infection with the varicella-zoster virus (VZV), is a rare but potentially severe condition with diverse clinical presentations. This case report highlights an instance where the mother developed a maculopapular rash seven days before delivery, indicating a possible transmission of VZV to the neonate. The patient's family history included recent diagnoses of herpes zoster and varicella among household members. On the second day of life, the newborn developed a discrete vesicular rash on an erythematous background, affecting the trunk and neck. Due to the unavailability of varicella zoster immunoglobulin (VZIG), intravenous immunoglobulin (IVIG) was administered along with a seven-day course of intravenous acyclovir. Despite the absence of VZIG, the combined treatment with IVIG and acyclovir proved effective in resolving the rash by the sixth day of life, without any ensuing complications. This case underscores the challenges of managing neonatal varicella in resource-limited settings and suggests that combination therapy may not prevent the occurrence of neonatal varicella but can mitigate serious complications and expedite clinical recovery.

## Introduction

The varicella-zoster virus (VZV), a double-stranded encapsulated DNA virus classified under the herpesvirus family (human herpesvirus type 3, HHV-3) [1], has an incubation period ranging from 10 to 23 days with an average of 14 days [2], preceding the appearance of the characteristic rash.

In the last trimester of pregnancy, congenital varicella is less likely, but the risk of disseminated varicella infection in the neonate (neonatal varicella) is elevated if the mother develops a varicella rash between seven days before and seven days after delivery [3], during which the mortality rate for the neonate can rise to 20% [4]. The mode of transmission to the infant could be in utero through the placenta, via ascending infection, or during passage through the birth canal.

Neonatal varicella is a rare condition, with an incidence of two to six cases per 100,000 live births per year [5], and it is a consequence of maternal VZV infection during pregnancy, presenting a complex clinical scenario for both mother and newborn. Varicella, commonly recognized as chickenpox, is a contagious viral illness associated with characteristic skin lesions and systemic symptoms. While maternal varicella infections are generally self-limiting, the neonatal population faces an increasing risk of severe morbidity and mortality when exposed to the virus during the perinatal period.

The severity of neonatal varicella ranges from mild manifestations to more severe complications, including varicella pneumonia, hepatitis, and meningoencephalitis, with the potential for fatal outcomes [6].

Hence, infants born to mothers who develop varicella within five days before delivery to two days after birth should promptly receive varicella-zoster immunoglobulin (VZIG). In the absence of VZIG, intravenous immunoglobulin (IVIG) is a suitable alternative. In addition, the immediate intervention involves the administration of intravenous acyclovir when lesions develop [7].

## Case presentation

A male baby was born to a 26-year-old mother at full term (39+ weeks) by cesarean section due to a maternal varicella infection. The mother had developed vesicular skin eruptions of chicken pox one week prior to delivery, following contact with an infected individual.

The baby was born with an Apgar score of 8/10 and 9/10 at the first and fifth minutes, respectively, with no dermatological lesions noted. On physical examination of the baby, he was not dysmorphic, was actively alert, responsive to stimuli, well perfused, not in respiratory distress, not cyanosed, and had good oral intake.

In the respiratory examination, he was not tachypneic, had no retraction, had good bilateral airway entry, and no add sounds. Abdominal examination showed a soft and lax abdomen, with no distention and no hepatosplenomegaly. Neurological examination showed an alert status, responsive to stimuli, with intact primitive reflexes (moro reflex, rooting reflex, sucking reflex, and grasp reflex) and no hypotonia, and all vital signs were within normal limits.

As the mother had developed the characteristic rash of chicken pox one week before childbirth, the decision was made to admit the newborn to a neonatal intensive care unit to manage any potential respiratory emergencies and to isolate him.

Based on the maternal history typical of chicken pox, a diagnosis of neonatal varicella was made, and despite the absence of a characteristic rash in the baby, an intrauterine infection was strongly suspected.

Unfortunately, due to VZIG unavailability, we used alternative treatments including IVIG at a dose of 500 mg/kg/day as a stat dose and IV acyclovir at a dose of 10 mg/kg/dose every eight hours for seven days, initiated on the baby’s first day of life.

**Figure 1:**
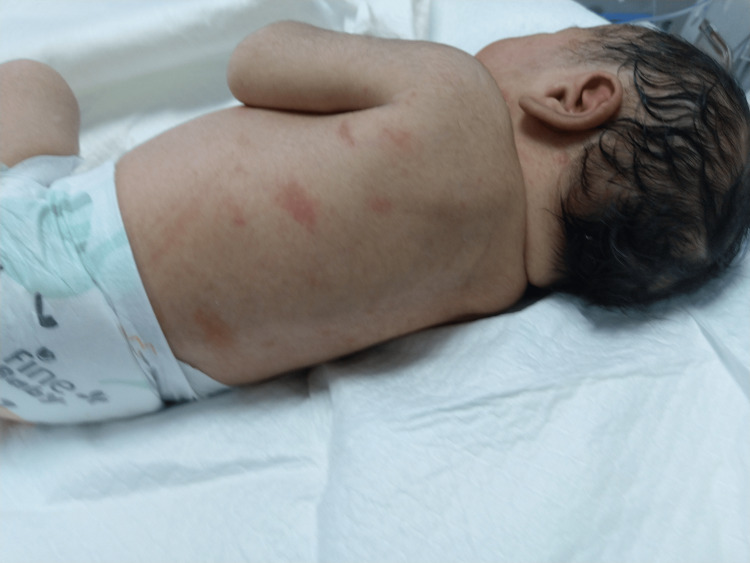
Small vesicle on an erythematous base on the second day of life on the posterior aspect of neck and multiple erythematous macules on the back, suggestive of erythema toxicum.

On the second day of life, the baby developed a vesicular rash on the posterior aspect of the neck and chest (Figure 1), which completely disappeared by the baby’s sixth day of life. The baby was discharged on his seventh day of life, when an acyclovir infusion of seven days was completed and a blood culture showed negative results, with normal full blood count and kidney and liver function tests.

Throughout the hospitalization, the baby remained afebrile, showed positive progress, maintained satisfactory oral intake, and sustained stable vital signs and normal neurological examination.

A scheduled follow-up appointment with a pediatrician was arranged for two weeks post-discharge. During the subsequent visit, the infant appeared in good health, had adequate feeding, and showed no signs of complications.

## Discussion

The initial documentation of disseminated varicella in newborns dates back to the late 19th century [8]. The frequency of varicella occurrence during pregnancy has been estimated to range from 0.1 to 0.7 per 1000 pregnancies, given that a substantial portion of the population, approximately 88 to 98%, possesses antibodies against VZV by the age of 20-40 years [9-11]. This high seroprevalence reflects widespread immunity within the reproductive-age population, influencing the relatively low incidence of varicella-related complications during pregnancy.

Neonatal varicella and congenital varicella are distinct manifestations of VZV infection during pregnancy, each presenting unique challenges and implications for both the mother and the newborn. Neonatal varicella refers to the occurrence of varicella in the newborn within the first 28 days of life, typically as a result of maternal infection near the time of delivery, and rarely encountered and treated even less [12]. By contrast, congenital varicella syndrome (CVS) arises when the fetus is exposed to VZV during the first 20 weeks of gestation, leading to a spectrum of congenital abnormalities. While both conditions involve maternal VZV infection during pregnancy, neonatal varicella primarily manifests as an acute infection in the newborn, with potential complications, such as pneumonia and disseminated disease. By contrast, CVS may result in structural anomalies affecting the skin, limbs, and central nervous system.

This disease is caused by the VZV. Transmission occurs through direct contact or by inhaling aerosols containing fluid from skin lesions, which originate from either acute varicella or zoster. In addition, the infection can be propagated through respiratory secretions that become aerosolized [13]. This virus could also be transmitted transplacentally, resulting in neonatal varicella. In such cases, the clinical diagnosis of neonatal varicella is established based on the observed symptoms and clinical presentation.

The clinical presentation of varicella includes an erythematous macular rash that quickly evolves into papules, vesicles, and pustules, resulting in a multiform appearance. Typically, this rash spreads in a centripetal pattern, starting from the central part of the body and extending to the face and extremities [14].

Maternal chickenpox around the time of delivery can pose a serious risk to newborns, often resulting in severe or even fatal illness. Despite the availability of VZIG in the USA, many European countries lack access to this therapy and require alternative preventive measures. Recent studies [15-16] suggest that a combination of IVIG and acyclovir (ACV) administered intravenously could be an effective strategy to prevent perinatal varicella. In a study involving 24 newborn infants whose mothers developed a varicella rash within 14 days before or after delivery, 15 infants identified as at-risk received IVIG prophylaxis (500 mg/kg) immediately after birth or post-natal contact, either alone or with intravenous ACV (5 mg/kg every eight hours) for five days, starting seven days after the onset of the maternal rash. The results were promising: among the four infants receiving IVIG alone, two developed clinical varicella, while none of the ten infants receiving both IVIG and ACV contracted the disease. In addition, one infant treated with ACV alone did not develop varicella vesicles. These findings highlight the potential of combined IVIG and ACV therapy as a viable preventive approach against neonatal varicella, particularly in regions where VZIG is not readily available.

No controlled clinical trials have assessed the efficacy of ACV in treating neonatal varicella. However, most experts endorse its use in managing the disease in newborns because ACV is one of the few antiviral medications proven effective against herpes viruses, with adequate pharmacokinetic and safety data for neonates. Recent studies indicate that current dosing regimens of ACV may not achieve therapeutic concentrations in the central nervous system, suggesting that more frequent dosing might be necessary for full-term neonates [17].

After the maternal diagnosis of chickenpox around term, an elective delivery might be postponed by five to seven days to allow the transfer of passive immunity to the neonate [18]. This delay provides the mother’s immune system additional time to produce antibodies against the varicella virus, which can then be transferred to the baby through the placenta. This transfer of antibodies helps to protect the newborn from the infection, reducing the risk of neonatal varicella, which can be severe. Thus, the strategic timing of delivery plays a crucial role in enhancing the newborn's immune defense against chickenpox.

The administration of IVIG to the newborn was initiated due to the unavailability of VZIG on the hospital's medication list, in addition to IV ACV. This decision was influenced by the findings of a prior study conducted by Huang et al. [15], where a combination of IVIG and IV ACV was successfully administered to newborns born to mothers with varicella rash within seven days before and five days after delivery, resulting in no occurrences of varicella. However, in the current case, the infant developed a rash despite receiving IVIG and ACV. Fortunately, no complications ensued post-administration of IVIG and ACV, leading to a complete recovery, and the baby was discharged without any residual issues. In summary, while the combination of IVIG and ACV may not have entirely prevented neonatal varicella in this instance, it proved effective in preventing serious complications and reducing the overall clinical duration of the illness.

## Conclusions

This case highlights the challenges of treating neonatal varicella in resource-limited settings. It suggests that while the combined use of IVIG and ACV may not completely prevent the disease in newborns, this medication combination could significantly lower the risk of severe complications and possibly shorten the duration of the illness. Consequently, despite its limitations, this approach may provide a critical advantage in managing neonatal varicella in challenging environments.
